# Carbonyl reductase identification and development of whole-cell biotransformation for highly efficient synthesis of (*R*)-[3,5-bis(trifluoromethyl)phenyl] ethanol

**DOI:** 10.1186/s12934-016-0585-5

**Published:** 2016-11-11

**Authors:** Kangling Chen, Kefei Li, Jian Deng, Baoqi Zhang, Jinping Lin, Dongzhi Wei

**Affiliations:** State Key Laboratory of Bioreactor Engineering, New World Institute of Biotechnology, East China University of Science and Technology, Shanghai, 200237 China

**Keywords:** (*R*)-[3,5-bis(trifluoromethyl)phenyl] ethanol, 3,5-bis(trifluoromethyl) acetophenone, Fusion-protein expression, Carbonyl reductase, Glucose dehydrogenase

## Abstract

**Background:**

(*R*)-[3,5-bis(trifluoromethyl)phenyl] ethanol [(*R*)-3,5-BTPE] is a valuable chiral intermediate for Aprepitant (Emend) and Fosaprepitant (Ivemend). Biocatalyzed asymmetric reduction is a preferred approach to synthesize highly optically active (*R*)-3,5-BTPE. However, the product concentration and productivity of reported (*R*)-3,5-BTPE synthetic processes remain unsatisfied.

**Results:**

A NADPH-dependent carbonyl reductase from *Lactobacillus kefir* (*Lk*CR) was discovered by genome mining for reduction of 3,5-bis(trifluoromethyl) acetophenone (3,5-BTAP) into (*R*)-3,5-BTPE with excellent enantioselectivity. In order to synthesize (*R*)-3,5-BTPE efficiently, *Lk*CR was coexpressed with glucose dehydrogenase from *Bacillus subtilis* (*Bs*GDH) for NADPH regeneration in *Escherichia coli* BL21 (DE3) cells, and the optimal recombinant strain produced 250.3 g/L (*R*)-3,5-BTPE with 99.9% *ee* but an unsatisfied productivity of 5.21 g/(L h). Then, four different linker peptides were used for the fusion expression of *Lk*CR and *Bs*GDH in *E. coli* to regulate catalytic efficiency of the enzymes and improved NADPH-recycling efficiency. Using the best strain (*E. coli*/pET-*Bs*GDH-ER/K(10 nm)-*Lk*CR), up to 297.3 g/L (*R*)-3,5-BTPE with enantiopurity >99.9% *ee* was produced via reduction of as much as 1.2 M of substrate with a 96.7% yield and productivity of 29.7 g/(L h).

**Conclusions:**

Recombinant *E. coli*/pET-*Bs*GDH-ER/K(10 nm)-*Lk*CR was developed for the bioreduction of 3,5-BTAP to (*R*)-3,5-BTPE, offered the best results in terms of high product concentration and productivity, demonstrating its great potential in industrial manufacturing of (*R*)-3,5-BTPE.

**Electronic supplementary material:**

The online version of this article (doi:10.1186/s12934-016-0585-5) contains supplementary material, which is available to authorized users.

## Background

Optically active alcohols are highly valuable chiral synthon pharmaceuticals and fine chemicals [1, 2]. (*R*)-[3,5-bis(trifluoromethyl) phenyl] ethanol [(*R*)-3,5-BTPE] is a key intermediate for the synthesis of neurokinin-1 receptor antagonists, such as Aprepitant (Emend) and Fosaprepitant (Ivemend), which are widely used in the treatment of chemotherapy-induced nausea and vomiting [3–5].

Asymmetric reduction of the prochiral ketone 3,5-bis(trifluoromethyl) acetophenone (3,5-BTAP) is an efficient and powerful way to produce highly optically active (*R*)-3,5-BTPE. Compared with conventional chemical synthesis via ruthenium-catalyzed transfer hydrogenation or oxazaborolidine-catalyzed borane reduction [6, 7], a biocatalyst-mediated reduction of 3,5-BTAP using microbial cells and various oxidoreductases has attracted more attention due to its excellent enantioselectivity, mild reaction conditions, few by-products and avoidance of residual metals. In recent years, the application of ketone reductases for synthesis of chiral alcohols was demonstrated on an industrial scale [8, 9]. Nevertheless, the large-scale biocatalytic production of (*R*)-3,5-BTPE remains difficult because asymmetric reduction of 3,5-BTAP to (*R*)-3,5-BTPE requires a biocatalyst with excellent anti-Prelog stereoselectivity, which is relatively rare in nature. To date, only five microbial strains, including *Lactobacillus kefir* [10], *Penicillium expansum* [11], *Leifsonia xyli* [12], *Microbacterium oxydans* [13], and *Trichoderma asperellum* ZJPH0801 [14], were reported for their abilities to reduce 3,5-BTAP to (*R*)-3,5-BTPE enantioselectively with >99% *ee*. However, these microbial cell-mediated bioreduction methods were successfully performed with conversion rates of 31–95% at relatively low substrate concentrations (less than 200 mM 3,5-BTAP), which restricted their industrial applications. Compared with the natural “producers” of (*R*)-3,5-BTPE, few ketone/carbonyl reductases have been discovered for synthesis of optically pure (*R*)-3,5-BTPE from 3,5-BTAP. Nevertheless, these enzyme-catalyzed reductions provide higher production. The crude recombinant ChKRED20 from *Chryseobacterium* sp. CA49 catalyzes the reduction of 150 g/L 3,5-BTAP to (*R*)-3,5-BTPE, with >99% conversion and >99.9% *ee* in 24 h [15], and the commercially available ketoreductase P1B2 from Codexis reduced 150 g/L 3,5-BTAP to (*R*)-3,5-BTPE with 98–99% conversion and >99% *ee* [16]. For these two enzyme-catalyzed reactions, the requirement of 30–40% (v/v) isopropanol as a co-substrate, as well as the addition of an expensive cofactor in the oxidized form of nicotinamide adenine dinucleotide phosphate [NAD(P)^+^], was necessary to regenerate the reduced cofactor. In terms of biocatalytic efficiency, the requirement for NAD(P)+ addition is disadvantageous. To overcome the problem of cofactor regeneration and to improve the reaction process, co-expression of two or more enzymes in a single cell was demonstrated as a promising and effective approach in many known processes [17, 18]. Specifically, the use of recombinant cells coupled with cofactor regeneration as catalysts can facilitate bio-redox reactions [19, 20]. Wang et al. reported that the whole cells of *Escherichia coli* expressing a mutant form of carbonyl reductase from *L. xyli* HS0904 (LXCAR-S154Y) reduced 1 M (256 g/L) 3,5-BTAP in the presence of 20% (v/v) isopropanol as co-substrate [21]. This reaction produced the desired (*R*)-3,5-BTPE with >99% *ee* but with an unsatisfactory 82.5% product yield. Recently, the addition of ionic liquid tetramethyl ammonium cysteine [N1,1,1,1][Cys] as a co-solvent in this reaction system enabled the reduction to proceed smoothly and increased the production yield up to 98.7% [22]. Although a high-product titer and productivity were obtained, the high price of ionic liquid and its problematic reusability, as well as the uncertain toxicity and potential environmental impact, restrict this bioreduction process for practical application. Therefore, there is great interest in searching for new carbonyl reductases with high enantioselectivity at high substrate concentrations and improving their application performance.

With the increased availability of public genome information, many putative carbonyl reductases can be obtained from GenBank (http://www.ncbi.nlm.nih.gov/genbank/). In this study, a NADPH-dependent carbonyl reductase from *L. kefir* (*Lk*CR) was discovered as a practical catalyst for (*R*)-3,5-BTPE synthesis by genome data mining. Generally, carbonyl reductases require nicotinamide adenine dinucleotide (NADH) or nicotinamide adenine dinucleotide phosphate (NADPH) as a cofactor for the reduction reactions. Because of the high cost of these cofactors, we used glucose dehydrogenase from *Bacillus subtilis* (*Bs*GDH) for in situ recycling of NAD(P)H for the asymmetric reduction of 3,5-BTAP by *Lk*CR. A whole-cell catalyst from *E. coli* co-expressing *Lk*CR and *Bs*GDH in tandem was constructed, and various parameters (reaction pH, reaction temperature, cell dosage, and substrate loading) from the whole-cell biotransformations were investigated. Furthermore, four different linker peptides were used for the fusion expression of *Lk*CR and *Bs*GDH. The best recombinant strain of *E. coli* (pET-*Bs*GDH-ER/K(10 nm)-*Lk*CR) was characterized for highly efficient production of (*R*)-3,5-BTPE at a high substrate load.

## Results and discussion

### Screening of oxidoreductases

A genome mining approach was used to search for carbonyl reductases that might be able to asymmetrically reduce 3,5-BTAP to the corresponding alcohol. In total, 60 known or putative carbonyl reductases were selected from the NCBI database and overexpressed in *E. coli* BL21 (DE3) cells. After testing their activities toward 3,5-BTAP using whole cells of *E. coli*, two carbonyl reductases were observed capable of reducing 3,5-BTAP to (*R*)-3,5-BTPE with excellent enantioselectivity, while two enzymes generated (*S*)-3,5-BTPE (Table 1). By comparing the conversion rate between the two (*R*)-3,5-BTPE-producing reductases at low-substrate concentration (50 mM), we achieved 97.6% conversion with CR-2 in 24 h, while 43.1% conversion was observed for CR-1 using the same concentration of biocatalyst (Table 1). Therefore, the carbonyl reductase CR-2 was chosen for further studies and was referred to as *Lk*CR.

**Table 1:** Screening of carbonyl reductases for 3,5-BTAP reduction

Enzyme	Sources of strains	Conversions (%)	ee (%)
CR-1	*Synechocystis* sp. *PCC 6803*	43.1	>99(R)
CR-2	*Lactobacillu kefir* DSM 20587	97.6	>99(R)
CR-3	*Lactobacillu kefir* DSM 20587	15.3	>99(S)
CR-4	*Gordonia* *polyisoprenivorans CCTT 137*	13.6	>99(S)

Substrate (50 mM), resting cells (50 g/L), and isopropanol [5% (v/v)] as co-substrate were mixed in PBS (100 mM, pH 7.0). The reaction was performed with shaking at 220 rpm and 30 °C for 24 h

Amino acid sequence alignment of the four carbonyl reductases (Additional file 1: Figure S1) revealed that both CR-1 **(**GenBank accession number: CP012832.1) and CR-2 (*Lk*CR, GenBank accession number: AY267012.1) exhibited excellent anti-Prelog stereoselectivity toward 3,5-BTAP, while sharing 33.9% sequence identity. CR-3 (GenBank accession number: EU877965.1) showed very low amino acid sequence identity (22.2%) with CR-2, thus, CR-2 and CR-3 were identified as different carbonyl reductases from *L. kefir*. CR-2 was firstly screened from *L. kefiri* DSM 20587 and characterized by Hummel et al. [23, 24]. This enzyme was identified as a NADPH dependent *R*-specific alcohol dehydrogenase and belonged to the short-chain dehydrogenase/reductase (SDR) family. It catalyzed the reduction of acetophenone and its derivatives (4-chloro-acetophene and 4-bromo-acetophene) to the corresponding (*R*)-alcohols. Several other aromatic and long-chain aliphatic secondary ketones aliphatic and aromatic ketones as well as β-keto esters were substrates for this enzyme. CR-3 was also isolated from *L. kefiri* DSM 20587, and was identified as a (*S*)-specific NADH-dependent alcohol dehydrogenase which belonged to the family of NAD(P)^+^-dependent medium-chain zinc-dependent alcohol dehydrogenases, group II. Zhu et al. reported that CR-3 also showed the reductive activity toward acetophenone, but producing (*S*)-alcohols. Compared to ketones, CR-3 preferred aldehydes as substrates [25]. Together with our study, *L. kefir* has at least two carbonyl reductases (CR-2 and CR-3) with reductive activity toward ketones but opposite enantioselectivity. Although the whole cells of *L. kefiri* DSM 20587 was used to reduce 3,5-BTAP to (*R*)-3,5-BTPE with >99% ee [10], (*S*)-3,5-BTPE producing enzyme was also existed in this strain, which was demonstrated in our study. Commercially available alcohol dehydrogenase (ADH) from *L. kefir* was used to transform 3,5-BTAP to (*R*)-3,5-BTPE with >99% ee in a very moderate yield [10], but very limited information about this enzyme was provided in this literature. It is hard to explain the relationship between this commercial ADH and CR-2.

### *Lk*CR characterization

**Fig. 1:**
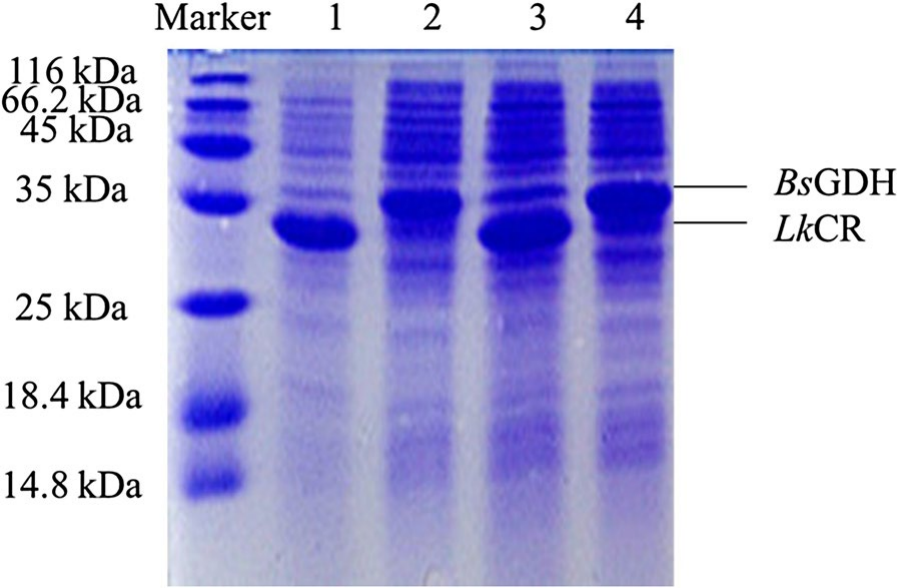
SDS-PAGE analysis of recombinant proteins. *Lane M*, protein markers; *lane 1*, crude extract of *E. coli*/pET-*Lk*CR; *lane 2*, crude extract of *E. coli*/pET-*Bs*GDH; *lane 3*, crude extract of *E. coli*/pET-*Lk*CR-*Bs*GDH; *lane 4*, crude extract of *E. coli*/pET-*Bs*GDH-*Lk*CR

**Fig. 2:**
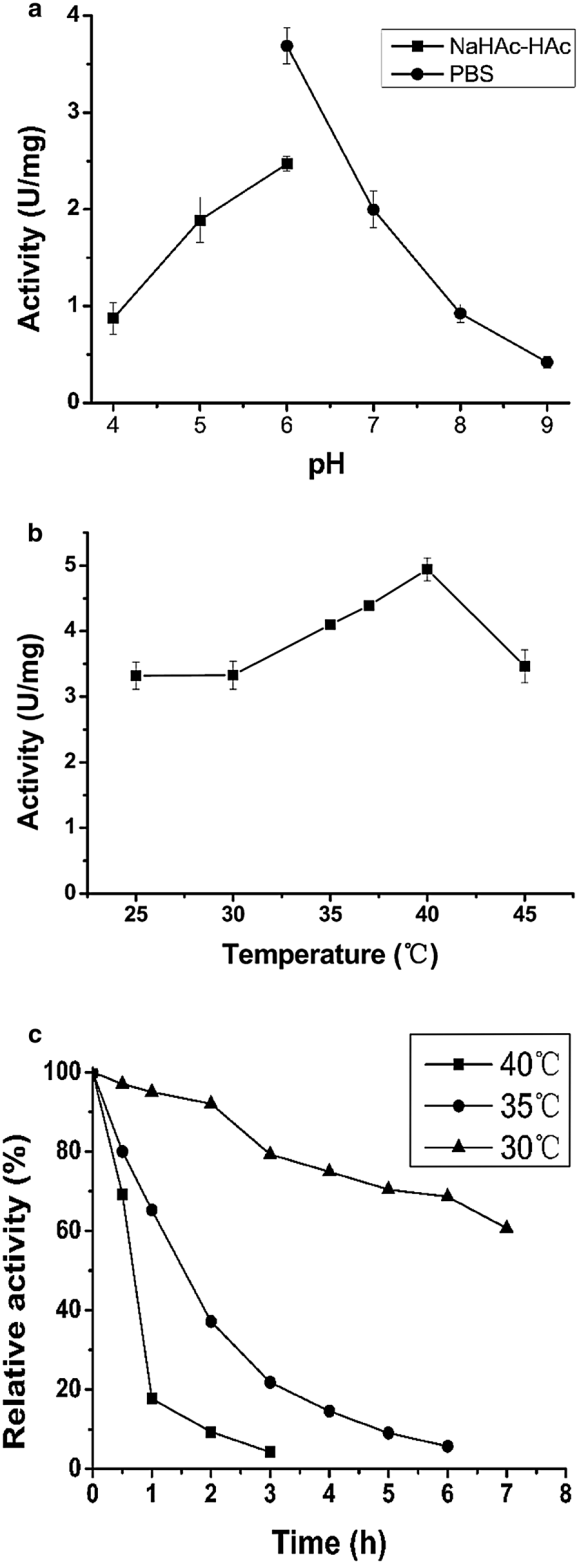
The effects of pH and temperature on the activity and thermostability of purified *Lk*CR. **a** Activity-pH profile; **b** activity-temperature profile; **c** thermostability at different temperatures

*Lk*CR containing an N-terminal His-tag was purified to homogeneity by nickel-affinity chromatography, and SDS-PAGE analysis of the crude extract demonstrated that most of the recombinant *Lk*CR existed in the soluble form at ~34 kDa (Fig. 1, lane 1). The specific activity of the purified enzyme was 6.1 U/mg toward 3,5-BTAP. Purified *Lk*CR only used NADPH instead of NADH as a coenzyme, indicating a NADPH-dependence. The optimal pH and temperature required for activity of purified *Lk*CR were 6.0 and 40 °C, respectively (Fig. 2a, b). Figure 2c showed that the half-life of purified *Lk*CR was only 39.4 and 87.2 min at 40 and 35 °C, respectively, but 582.8 min at 30 °C, indicating that purified *Lk*CR was relatively stable at 30 °C.

### Asymmetric synthesis of (*R*)-3,5-BTPE from 3,5-BTAP by whole cells of *E. coli* co-expressing *Lk*CR and *Bs*GDH

Given that glucose as the substrate of glucose dehydrogenase (GDH) is inexpensive and that GDH can regenerate both NADH and NADPH, GDH is the most commonly used dehydrogenase for coenzyme regeneration. Here, *Bs*GDH was used for NADPH regeneration to promote *Lk*CR reduction of 3,5-BTAP. Previously, *Bs*GDH was successfully applied in multiple NADH-dependent and NADPH-dependent biotransformations [26, 27]. Compared with isolated enzymes, preparation of whole-cell catalysts is easier, more cost effective, and results in enzymes exhibiting higher degrees of stability. Therefore, *Lk*CR and *Bs*GDH were co-expressed in *E. coli* BL21 (DE3) cells in tandem, and the whole cells of *E. coli* containing the recombinant proteins expressed by the pET-*Lk*CR-*Bs*GDH plasmid were employed as biocatalysts for the reduction of 3,5-BTAP.

The functional expression of both enzymes was determined by measuring their activities in cell-free extracts. *Lk*CR activity toward 3,5-BTAP was 73.0 U/g wet cells, which was significantly higher than the activity of *Bs*GDH using glucose as a substrate (12.9 U/g wet cells). SDS-PAGE analysis of protein extracts from *E. coli*/pET-*Lk*CR-*Bs*GDH cells showed that the expression of *Lk*CR placed upstream was relatively higher than that of *Bs*GDH positioned downstream (Fig. 1, lane 3). In contrast, we observed lower *Lk*CR expression levels (Fig. 1, lane 4) accompanied by lower levels of activity (25.8 U/g wet cells) in *E. coli*/pET-*Bs*GDH-*Lk*CR, with *Lk*CR positioned downstream and *Bs*GDH positioned upstream, resulting in decreased conversion of 3,5-BTAP. Consequently, *E. coli*/pET-*Lk*CR-*Bs*GDH was chosen for further research.

To achieve a higher production titer, we optimized the biocatalytic conditions necessary for producing (*R*)-3,5-BTPE from 3,5-BTAP using whole cells of *E. coli*/pET-*Lk*CR-*Bs*GDH. The influence of the reaction temperature was determined in reaction mixtures containing 250 g (wet)/L whole cells, 300 mM 3,5-BTAP, 450 mM glucose, and 100 mM PBS (pH 7.0). As shown in Table 2, over a range of 20–35 °C, we observed the highest yield (97.7%) at 28 °C. Only minor yield changes were observed at temperatures between 25 and 30 °C, but at higher temperatures, product yield decreased dramatically. The *ee* of (*R*)-3,5-BTPE was not sensitive to temperature changes, and *ee* values remained at >99% under all conditions. In order to determine the optimum pH necessary for the bioconversion of 3,5-BTAP, the reaction was carried out at different pH values ranging from 4.0 to 7.0 at 28 °C.While pH significantly affected 3,5-BTAP bioconversion, it had no effect on the (*R*)-3,5-BTPE *ee* value (Table 2). The highest (*R*)-3,5-BTPE production was detected at pH 5.5; however, at pH values <5.5, 3,5-BTAP conversion decreased sharply. Moreover, high ee values (>99%) were obtained under different pH conditions.

**Table 2:** The effect of temperature and pH on asymmetric (*R*)-3,5-BTPE synthesis by recombinant *E.coli*  cells co-expressing LkCR and BsGDH

	Reaction condition	Yield (%)	ee (%)-(R)-configuration
Temperature(°C)^a^	20	86.7 ± 1.6	>99
	25	94.5 ± 2.4	>99
	28	97.7 ± 0.5	>99
	30	95.7 ± 0.6	>99
	35	82.8 ± 0.9	>99
pH^b^	4.0	52.3 ± 0.4	>99
	4.5	75.1 ± 0.6	>99
	5.0	80.0 ± 0.2	>99
	5.5	97.3 ± 1.6	>99
	6.0	87.4 ± 1.8	>99
	6.5	84.2 ± 1.2	>99
	7.0	82.4 ± 0.6	>99

Reaction conditions: 300 mM substrate, 450 mM glucose, 250 g/L wet cells, and incubation for 30 h with shaking at 220 rpm

^a^Reaction was performed in 100 mM PBS buffer (pH 7.0)

^b^Temperature was 28 °C; pH 4.0–6.0 (0.2 M, NaAc–HAc), pH 6.0–7.0 (0.1 M Na_2_HPO_4_–NaH_2_PO_4_)

**Fig. 3:**
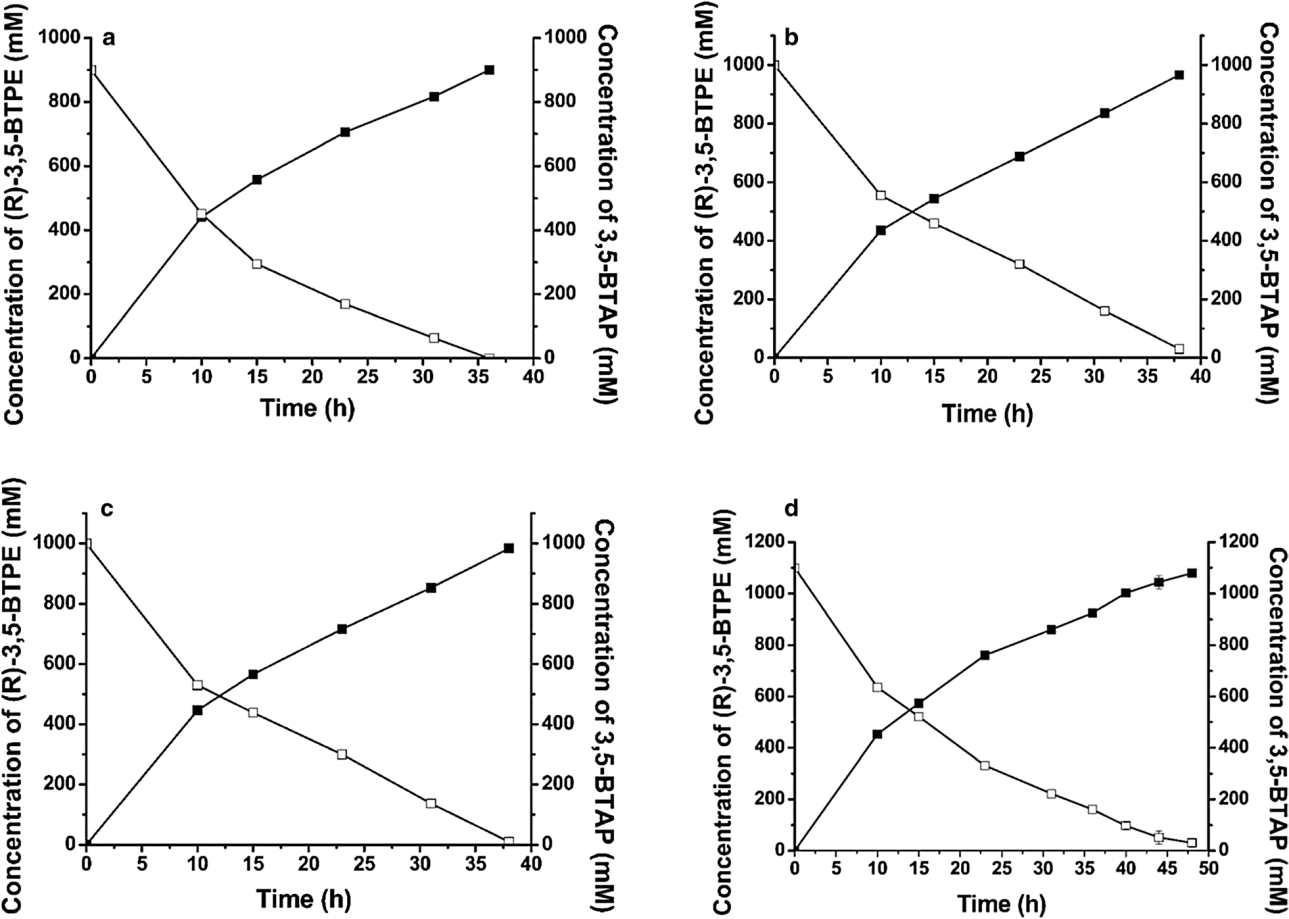
The (*R*)-3,5-BTPE concentration formed in the presence of different biocatalysts. **a** 3, 5-BTAP (900 mM) with 350 g/L wet cells; **b** 1 M 3, 5-BTAP with 350 g/L wet cells; **c** 1 M 3, 5-BTAP with 375 g/L wet cells; **d** 1.1 M 3,5-BTAP with 375 g/L wet cells. Concentration of 3,5-BTAP (*open square*) and (*R*)-3,5-BTPE (*filled square*) are shown

To determine the biocatalyst dosage necessary for optimal bioconversion, we examined the effect of cell concentration on 3,5-BTAP reduction. A ratio of 1.5:1 glucose to 3,5-BTAP was added to the reactions based on its producing the highest (*R*)-3,5-BTPE yield (Additional file 2: Figure S2), and the reactions were performed at 28 °C and pH 5.5. As shown in Fig. 3a, when 3,5-BTAP concentration in the reaction was <900 mM, 350 g (wet)/L cells were sufficient to achieve a 100% conversion of 3,5-BTAP to (*R*)-3,5-BTPE within 36 h, with an excellent (*R*)-3,5-BTPE yield of >99%. The substrate was increased stepwise to 1 M, resulting in a 96.1% yield over a reaction time of 38 h (Fig. 3b). When the cell concentration was increased to 375 g (wet)/L, conversion rate was slightly increased to 98.3% within 38 h, and the *ee* of (*R*)-3,5-BTPE was 99.9% (Fig. 3c). A further increase in substrate to 1.1 M resulted in 96.9% conversion to (*R*)-3,5-BTPE [1066.23 mM (275.24 g/L)] within 48 h, with an *ee* value of 99.9% and a productivity rate of 5.73 g/(L h) (Fig. 3d). During the reaction, we observed that the increased viscosity of the reaction system due to high cell concentrations (375 g/L) and increased glucose loading (1.65 mM) led to mass-transfer limitations, which affected catalytic efficiency.

### Expression of *Lk*CR and *Bs*GDH fusion proteins enhanced (*R*)-3,5-BTPE production

#### Expression of *Lk*CR and *Bs*GDH fusion proteins in *E. coli* BL21 cells

The co-expression of multiple fusion proteins can increase protein solubility and also result in a multifunctional enzyme [28]. Previous studies indicated that enhancing the spatial proximity of enzymes through the creation of fusion constructs could regulate catalytic efficiency and enhance product synthesis in multi-enzyme reactions [25, 29, 30, 31]. The common and easiest approach to construct a fusion enzyme is to fuse the sequentially acting enzymes end to end by a linker peptide. In addition to the necessity for an appropriate amino acid composition of the individual enzymes, the selection of the linker peptide is particularly important in the construction of fusion enzymes. The folding of linker peptides could have significant effect on the folding of macromolecules [32]. The linker sequence and length would limit the stability, flexibility, oligomeric state and solubility of the fusion protein and consequently affect its function or lead to expression failure [33]. Unfortunately, there are no reliable selection criteria or programs available for use in linker design, due to the lack of limited understanding of sequence-structure correlation for many linker peptides of various protein families. Most current linker selection is still largely dependent on intuition and test. In this study, fusion constructs containing both *Lk*CR and *Bs*GDH with different linker peptides were created, and the whole cells of *E. coli* expressing these variants were used to catalyze the conversion of higher concentrations of 3,5-BTAP.

**Fig. 4:**
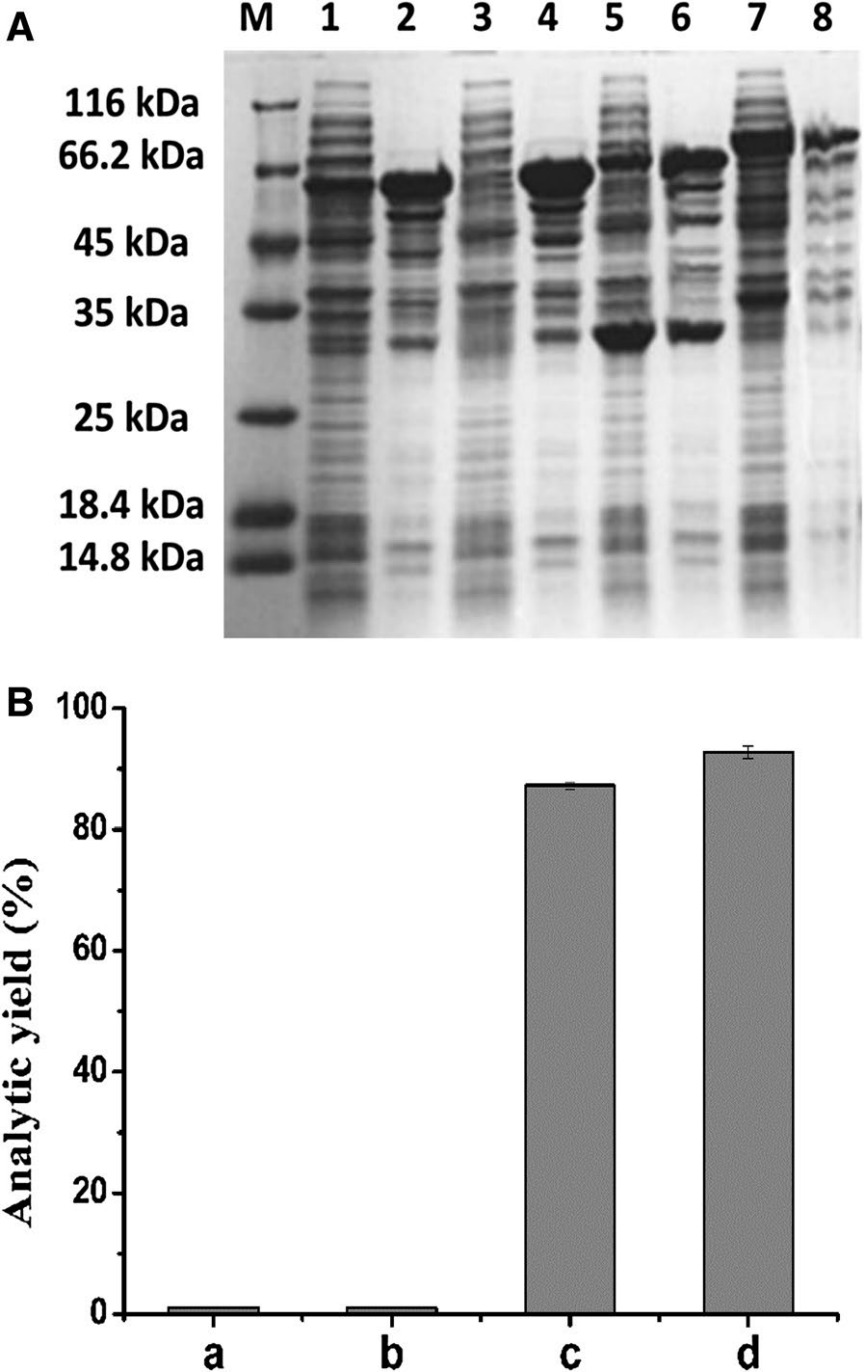
**A** SDS-PAGE analysis of the fusion expression of *Lk*CR and *Bs*GDH. *Lane M*, protein marker; *lane 1*, soluble pET-*Bs*GDH-(GGGGS)_3_-*Lk*CR protein; *lane 2*, insoluble pET-*Bs*GDH-(GGGGS)_3_-*Lk*CR protein; *lane 3*, soluble pET-*Bs*GDH-(EAAAK)_3_-*Lk*CR protein; *lane 4*, insoluble pET-*Bs*GDH-(EAAAK)_3_-*Lk*CR protein; *lane 5*, soluble pET-*Bs*GDH-ER/K(5 nm)-*Lk*CR protein; *lane 6*, insoluble pET-*Bs*GDH-ER/K(5 nm)-*Lk*CR protein; *lane 7*, soluble pET-*Bs*GDH-ER/K(10 nm)-*Lk*CR protein; *lane 8*, insoluble pET-*Bs*GDH-ER/K(10 nm)-*Lk*CR protein. **B** Production of (*R*)-3,5-BTPE in *E. coli* following the expression of fusion enzymes with four linkers. *a* pET-*Bs*GDH-(GGGGS)_3_-*Lk*CR, *b* pET-*Bs*GDH-(EAAAK)_3_-*Lk*CR, *c* pET-*Bs*GDH-ER/K(5 nm)-*Lk*CR, and *d* pET-*Bs*GDH-ER/K(10 nm)-*Lk*CR. Reactions were performed in the presence of 200 mM substrate, 300 mM glucose, and 50 g/L wet cells for 30 h at pH 5.5 with shaking at 220 rpm

To date, a large number of peptides have been used as linkers for construction of fusion enzymes. Of these, the flexible linkers (GGGGS)_*n*_ (usually n ≤ 6) are often used and can provide enzyme flexibility for catalysis domain separation [34, 35]. The typical rigid α-helical-forming linker (EAAAK)*n* (*n* ≤ 6) is utilized to link two domains of fusion enzymes by controlling a distance between them [36–38]. The long chain rigid linker peptide ER/K (5, 10 nm) had good effect for the expression of fusion protein [39, 40]. In the present study, four linker peptides (GGGGSGGGGSGGGGS, EAAAKEAAAKEAAAK, 5 and 10-nm rigid α-helical ER/K motifs) were used to fuse *Lk*CR and *Bs*GDH following linker attachment to the N-terminal region of *Lk*CR. This site was chosen for linker attachment based on its flexibility according to crystal-structure analysis of *Lk*CR (PDB ID: 4RF2) and its predicted limited effect on enzyme spatial structure. The four recombinant plasmids [pET-*Bs*GDH-(GGGGS)_3_-*Lk*CR, pET-*Bs*GDH-(EAAAK)_3_-*Lk*CR, pET-*Bs*GDH-ER/K(5 nm)-*Lk*CR, and pET-*Bs*GDH-ER/K(10 nm)-*Lk*CR) were transformed into *E. coli* BL21 (DE3) cells, and the recombinant proteins were expressed and purified. Each of the four *Lk*CR-*Bs*GDH fusion proteins migrated as a band indicating approximate sizes of 62, 62, 64 and 70 kDa by SDS-PAGE (Fig. 4A), which were in agreement with molecular weights predicted from the gene sequences. Incorporation of the (GGGGS)_3_ or (EAAAK)_3_ linker peptides resulted in the fusion proteins being insoluble (Fig. 4A, lanes 1–4), while the ER/K(5 nm) and ER/K(10 nm) linker peptides resulted in the expression of soluble forms of the fusion proteins (Fig. 4A, lanes 5–8), with most of the ER/K(10 nm) fusion protein being expressed in the soluble form.

Further comparison of (*R*)-3,5-BTPE production was performed among the resulting strains. At the substrate concentration of 200 mM, few (*R*)-3,5-BTPE could be produced by the *E. coli* strains with pET-*Bs*GDH-(GGGGS)_3_-*Lk*CR and pET-*Bs*GDH-(EAAAK)_3_-*Lk*CR due to the low soluble expression and very low activity of *Bs*GDH; whereas, 87.3 and 92.8% yields were observed for *E.coli*/pET-*Bs*GDH-ER/K(5 nm)-*Lk*CR and *E. coli*/pET-*Bs*GDH-ER/K(10 nm)-*Lk*CR (Fig. 4B). For *E. coli*/pET-*Bs*GDH-(GGGGS)_3_-*Lk*CR and *E. coli*/pET-*Bs*GDH-(EAAAK)_3_-*Lk*CR, the activity of *Lk*CR were 36.8 and 34.3 U/g wet cells, respectively, but *Bs*GDH activities almost could not be detected. As a result, the cofactor could not be regenerated. For *E. coli*/pET-*Bs*GDH-ER/K(10 nm)-*Lk*CR, the activity of *Lk*CR and *Bs*GDH were 80.4 U and 7.2 U per gram wet cells, respectively. In contrast, lower activity (75.8 U/g _(wet cells)_) of *Lk*CR and lower activity (6.8 U/g _(wet cells)_) of *Bs*GDH were observed in *E. coli*/pET-*Bs*GDH-ER/K(5 nm)-*Lk*CR. Consequently, the whole cells of *E. coli*/pET-*Bs*GDH-ER/K (10 nm)-*Lk*CR showed higher yield of (*R*)-3,5-BTPE than that by *E. coli*/pET-*Bs*GDH-ER/K(5 nm)-*Lk*CR. Therefore, *E. coli*/pET-*Bs*GDH-ER/K(10 nm)-*Lk*CR was chosen for asymmetric synthesis of (*R*)-3,5-BTPE.

#### Asymmetric synthesis of (*R*)-3,5-BTPE from 3,5-BTAP by whole cells of the recombinant *E. coli*/pET-*Bs*GDH-ER/K(10 nm)- *Lk*CR

**Fig. 5:**
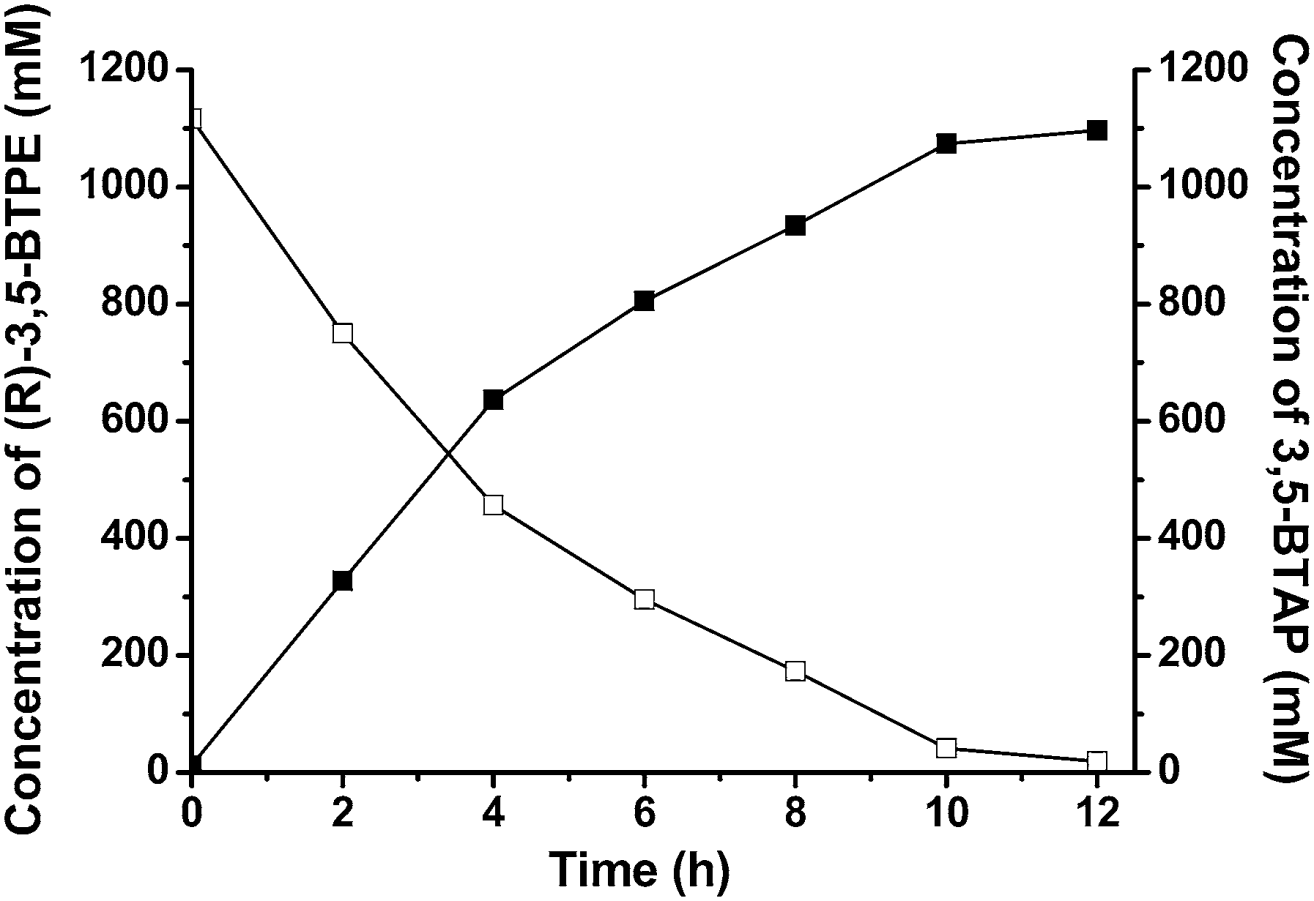
Bioconversion of 3, 5-BTAP to (*R*)-3,5-BTPE by whole cells of *E. coli/*pET-*Bs*GDH-ER/K(10 nm)-*Lk*CR. Concentrations of 3,5-BTAP (*open square*) and (*R*)-3,5-BTPE (*filled square*) are shown

After optimizing the reaction conditions (Additional file 3: Figure S3), a ratio of 1:1 glucose to 3,5-BTAP produced 1095.9 mM (282.9 g/L) of (*R*)-BTPE with >99% *ee* via reduction of 1.12 M 3.5-BTAP within 12 h by 300 g (wet)/L cells of *E. coli*/pET-*Bs*GDH-ER/K(10 nm)-*Lk*CR, resulting in a 98.2% yield and a productivity rate of 23.6 g/(L h) (Fig. 5). Compared to *E. coli*/pET-*Lk*CR-*Bs*GDH, *E. coli*/pET-*Bs*GDH-ER/K(10 nm)-*Lk*CR exhibited a higher catalytic efficiency and enhanced (*R*)-BTPE production at decreased cell concentrations and glucose loading, as well as shorter reaction time. The close spatial proximity of *Lk*CR and *Bs*GDH by virtue of the construction of the fusion-enzyme variant effectively regulated catalytic efficiency and improved NADPH-recycling efficiency.

To effectively synthesize (*R*)-3,5-BTPE at the gram scale, asymmetric reduction of 1.19 M 3,5-BTAP by *E. coli*/pET-*Bs*GDH-ER/K(10 nm)-*Lk*CR was performed in a 10-mL volume. Up to 297.3 g/L (*R*)-3,5-BTPE with >99.9% *ee* was produced by 300 g (wet)/L cells within 10 h, resulting in a 96.7% yield and a productivity rate of 29.7 g/(L h). Several microbial strains or alcohol dehydrogenases were reported to asymmetrically catalyze the reduction 3,5-BTAP to (*R*)-3,5-BTPE. Of these, four bioprocesses indicating potential for industrial-scale application were reported with a relative high production of (*R*)-3,5-BTPE (Table 3). Compared with the processes catalyzed by the immobilized ketoreductase P1B2 or the lyophilized powder of the crude recombinant enzyme (ChKRED20), the whole cells of recombinant *E. coli* strain expressing carbonyl reductase LXCAR-S154Y gave the higher product titer and productivity. To the best of our knowledge, the highest production of (*R*)-3,5-BTPE was described by Wang et al. [22]. With ionic liquid as a co-solvent in reaction system, 252.7 g/L (*R*)-3,5-BTPE with >99.9% *ee* was produced from 1 M 3,5-BTAP within 12 h, giving a yield of 98.7% and a productivity of 21.1 g/(L h). By contrast, *E. coli*/pET-*Bs*GDH-ER/K(10 nm)-*Lk*CR mediated 3,5-BTAP reduction in present study exhibited the highest production titer (297.3 g/L) and productivity rates [29.7 g/(L h)].

**Table 3:** Comparison of the bioprocesses associated with (*R*)-3,5-BTPE production via 3,5-BTAP reduction

Enzyme/strain	Substrate concentration (mM)	Production concentration (g/L)	Yield (%)	Time (h)	ee (%)	Reference
Ketoreductase P1B2 from Codexis	586	145.50	97	24	>99.9 (R)	[16]
ChKRED20 from *Chryseobacterium* sp.CA49	586	139.168	92	24	>99.9 (R)	[29]
Mutant LXCAR-S154Y from *Leifsonia xyli* HS0904	1000	212.965	82.5	12	>99.9 (R)	[41]
Mutant LXCAR-S154Y in ionic liquid	1000	252.7	98.7	12	>99.9 (R)	[22]
*Lk*CR from *Lactobacillus kefir* fused with GDH	1191	297.27	96.7	10	>99.9 (R)	This study

## Conclusions

The NADPH-dependent carbonyl reductase from *L. kefir, Lk*CR, was discovered to have excellent enantioselectivity for 3,5-BTAP. *E. coli* strains overexpressing *Lk*CR and *Bs*GDH were developed via co-expressing and fusion expressing, and each was employed for 3,5-BTAP reduction in the presence of high substrate loading (0.9–1.2 M). Among these variants, *E. coli*/pET-*Bs*GDH-ER/K(10 nm)-*Lk*CR exhibited the highest productivity, with (*R*)-3,5-BTPE concentrations of 297.3 g/L at a 96.7% yield, an excellent *ee* value (>99.9%), and a high productivity rate [29.7 g/(L h)]. These results demonstrated that the developed biocatalytic process is scalable and has strong potential for the industrial-scale preparation of (*R*)-3,5-BTPE.

## Methods

### Chemicals

3,5-BTAP, (*R*)-3,5-BTPE, NADPH, and NADP^+^ were purchased from TCI (Tokyo, Japan), J&K (Shanghai, China), Roche (Basel, Switzerland) and Roche, respectively. Other chemicals involved were analytical grade.

### Cloning, expression, and purification of oxidoreductases

Carbonyl reductase genes used for screening were selected from the NCBI database (http://www.ncbi.nlm.nih.gov). Genomic DNA was extracted and purified using a TaKaRa MiniBEST Bacterial Genomic DNA Extraction Kit Ver.2.0 (TaKaRa, Beijing, China). The DNA fragment of the carbonyl reductase gene was amplified and double digested using *BamH*I and *Xho*I, and then inserted into the expression vector pET-28a (Novagen, Shanghai, China). The resulting plasmid was transformed into *E. coli* BL21 (DE3) cells cultured at 20 °C in lysogeny broth medium (1% NaCl, 1% peptone, and 0.5% yeast extract) containing 0.5 mM kanamycin. When the optical density at 600 nm of the culture reached 0.6–0.8, isopropyl β-D-1-thiogalactopyranoside (IPTG) was added to a final concentration of 0.1 mM, and cultivation was continued at 20 °C for another 14 h.

### Purification and characterization of *Lk*CR

Cells were harvested by centrifugation (8000*g* for 10 min) at 4 °C, washed twice with 20 mM sodium phosphate buffer (PBS, pH 7.4), and subsequently disrupted with an ultrasonic oscillator (JY92-II; Scientz Biotech. Co., Ltd., Ningbo, China). The cell lysate was removed by centrifugation (20 min at 10,000*g* rpm) at 4 °C, and the supernatant was loaded onto a 5 mL Ni–NTA FF column (GE Healthcare, Beijing, China), which was equilibrated with 20 mM imidazole buffer (pH 7.4) with saline. Proteins were eluted with an increasing gradient of 20 mM to 500 mM imidazole buffer (pH 7.4) with saline at a flow rate of 1 mL/min. The fractions containing the target protein were collected and dialyzed against 20 mM PBS (pH 7.4) for desalting. The sample was concentrated and stored at 4 °C, and sodium dodecyl sulfate polyacrylamide gel electrophoresis (SDS-PAGE) was used to verify *LkCR* expression and purification.

The activity of NADPH-dependent *LkCR* was assayed by measuring the change in absorbance at 340 nm according to NADPH oxidation or NADP^+^ reduction using an ultraviolet (UV)/visible spectrophotometer (Ultrospec 2100 pro, Amersham Biosciences, Piscataway, NJ, USA). A molar extinction coefficient of 6.22 mM/cm for NADPH was used for the calculation [42]. The reaction mixture (0.2 mL) for the enzyme assay consisted of 5 μL purified enzyme, 0.2 mM NADPH, and 2.5 mM 3,5-BTAP in 100 mM PBS (pH 6.0). One unit of NADPH-dependent carbonyl reductase activity was defined as the amount of enzyme that consumed l mol/min NADPH. The protein concentration was determined by the Lowry procedure using bovine serum albumin as the standard [43].

The optimum pH of *LkCR* was determined in sodium acetate-acetic acid (pH 4.0–6.0) and PBS (pH 6.0–9.0) buffers at final ionic concentrations of 100 mM. The optimum temperature was determined by testing at 25 °C to 45° under standard conditions. Thermal stability was determined by incubating the purified enzyme at 30, 35, or 40 °C followed by measuring residual enzyme activity.

### Co-expression of *Lk*CR and *Bs*GDH in *E. coli*

A pET-28a plasmid containing the *Lk*CR and *Bs*GDH genes under the control of an individual ribosome-binding site (RBS) region and a common T7 promotor was constructed. The RBS-*Bs*GDH gene was obtained from the pET28a- *Bs*GDH vector following amplification using primers P1 (5′-CCCAAGCTTGAAGGAGATATACCATGG-3′) containing a *Hind*III-restriction site and P2 (5′-CCGCTCGAGTTAACCGCGGCCTGCCTG-3′) containing a *Xho*I-restriction site. Following restriction digest of the products, the DNA segment was cloned into the *Hind*III/*Xho*I site of the pET28a- *Lk*CR vector to construct the recombinant pET-*Lk*CR-*Bs*GDH plasmid. Similarly, the RBS-*Lk*CR gene was acquired from the pET28a- *Lk*CR vector using primers P3 (5′-CCCAAGCTTGAAGGAGATATACCATGG-3′) containing a *Hind*III-restriction site and P4 (5′-CCGCTCGAGTTATTGAGCAGTGTATCC-3′) containing a *Xho*I-restriction site, and the digested product was ligated into the pET28a-*Bs*GDH vector to construct the recombinant pET-*Bs*GDH-*Lk*CR plasmid. The resulting recombinant plasmids were transformed into *E. coli* BL21 (DE3) cells.

### Fusion expression of *Lk*CR and *Bs*GDH in *E. coli*

The *Lk*CR and *Bs*GDH genes were spliced together using the splicing by overlap extension polymerase chain reaction method (SOE-PCR), which incorporated four different linker peptides **(**GGGGSGGGGSGGGGS, EAAAKEAAAKEAAAK, KAKLKEEEERKQREEEERIKRLEELAKRKEEERK, and EEEEKKKQQEEEAERLRRIQEEMEKERKRREEDEERRRKEEEERRMKLEMEAKRKQEEEERKKREDDEKRKKK) [39, 40] at the *Lk*CR N-terminus. Each fusion-PCR fragment was digested with *BamH*I/*Xho*I and ligated into similarly digested sites of pET-28a vectors, resulting in four plasmids: pET-*Bs*GDH-GGGGS-*Lk*CR, pET-*Bs*GDH-EAAAK-*Lk*CR, pET-*Bs*GDH-ER/K(5 nm)-*Lk*CR, pET-*Bs*GDH-ER/K(10 nm)-*Lk*CR (Additional file 4: Table S1). The resulting plasmids were transformed into *E. coli* BL21 (DE3) cells. The primers involved in the SOE-PCR are listed in Additional file 5: Table S2.

### Reduction of 3,5-BTAP to (*R*)-3,5-BTPE using whole cells

#### Preparation of whole-cell catalysts

Cultivation of *E. coli* BL21 (DE3) cells carrying the recombinant plasmid was performed in manner similar to described earlier; however, in this study, IPTG to a final concentration of 0.2 mM was added. After cultivation, induced cells were harvested by centrifugation (8000 rpm for 10 min) at 4 °C. Wet cells were washed twice in 20 mM PBS (pH 7.4) before further use.

#### Reaction conditions using *E. coli* co-expressing recombinant *Lk*CR and *Bs*GDH

For the reaction at low substrate concentrations (<500 mM), a 1-mL reaction system, including 300 mM 3,5-BTAP, 450 mM glucose as co-substrate, and 250 g/L wet cells, was used. The mixtures were incubated at 28 °C with shaking at 220 rpm for 30 h.

For the reaction at high substrate concentration (>500 mM), many parallel experiments at 1 mL were conducted, with each containing 0.4 M sodium acetate-acetic acid buffer (pH 5.5), 500 mM to 1.1 M 3,5-BTAP, glucose (at a 1.5:1 ratio of glucose to substrate concentration), and 0.3–0.375 g wet cells. The pH was adjusted from 5.0 to 5.5 using NaOH during the reaction. The mixtures were incubated at 28 °C with shaking at 220 rpm, and samples were collected at regular intervals.

#### Reaction conditions using *E. coli* fusion-expressing recombinant *Lk*CR and *Bs*GDH

Multiple parallel experiments at 1 mL were conducted, with each containing 0.4 M sodium acetate-acetic acid buffer (pH 5.5), 1.1–1.2 M 3,5-BTAP, glucose (at a 1:1 ratio of glucose to substrate concentration), and 0.3 g wet cells. The pH was adjusted from 5.0 to 5.5 using NaOH during the reaction. The mixtures were incubated at 28 °C with shaking at 220 rpm, and samples were collected at regular intervals.

Other reaction conditions, including reaction temperatures, initial pH, and glucose concentrations, were outlined in the text or tables.

#### Preparative synthesis of (*R*)-3,5-BTPE

The preparative-scale bioreduction was carried out in a round-bottomed flask (working volume: 20 mL). The reaction mixture contained 0.4 M sodium acetate-acetic acid buffer (pH 5.5), 300 g/L *E. coli Bs*GDH-ER/K- *Lk*CR wet cells, 1.2 M 3,5-BTAP, and 1.2 M glucose. The pH was adjusted from 5.0 to 5.5 using NaOH during the reaction using a pH automatic regulator (Chroma, Ningbo, China). The mixtures were incubated at 28 °C with shaking at 220 rpm, and samples were collected at regular intervals.

### Analytical methods

Glucose concentration was measured using a biosensor analyzer (SBA-40D; Institute of Biology, Shandong Province Academy of Sciences, China). 3,5-BTAP and (*R*)-3,5-BTPE concentrations were measured by high-performance liquid chromatography (HPLC; Agilent 1260; Agilent Technologies, Santa Clara, CA, USA) equipped with a Zorbax extend-C18 column (250 × 4.6 mm; Agilent Technologies). HPLC was performed with a 75:25 ratio of mobile phases A (water) and B (methanol) at 25 °C at a flow rate of 1.0 mL/min. The UV detection wavelength was 210 nm, and 3,5-BTAP and (*R*)-3,5-BTPE retention times were 7.5 and 6.75 min (Additional file 6: Figure S4), respectively. Enantiomeric excess of (*R*)-3,5-BTPE was measured by HPLC equipped with a Chiralcel OD-H column (25 × 0.46 cm; Daicel Co., Osaka, Japan), eluted with hexane/isopropanol [98:2 (v/v)] at a flow rate of 1.0 mL/min at 40 °C, and detected at 210 nm. Retention times of *R*- and *S*-3,5-BTPE were 8.0 and 9.2 min, respectively (Additional file 7: Figure S5).

## Additional files

**Additional file 1: Figure S1.** Amino acid sequence alignment of CR-1 (GenBank accession number: CP012832.1), CR-2 (AY267012.1), CR-3 (EU877965.1), and CR-4 (CP003119.1). The alignment was performed with the program DNAMAN (http://www.lynnon.com/). Gaps in the aligned sequences are indicated by dots. Highly similar residues are colored in red and framed in blue, while identical residues are in white and on a red background.

**Additional file 2: Figure S2.** The effect of glucose concentration on the asymmetric synthesis of (*R*)-3,5-BTPE using *E. coli*/pET-*Bs*GDH-*Lk*CR. Reaction conditions: 300 mM substrate, 330-540 mM glucose, 250 g/L wet cells, and incubated at 28°C for 24 h at pH 5.5 with shaking at 220 rpm.

**Additional file 3: Figure S3.** The effect of glucose concentration on the asymmetric synthesis of (*R*)-3,5-BTPE using *E. coli/*pET-*Bs*GDH-ER/K(10 nm)-*Lk*CR. Reaction conditions: 1200 mM substrate, 1200-1680 mM glucose, 300 g/L wet cells, and incubated at 28°C for 24 h at pH 5.5 with shaking at 220 rpm.

**Additional file 4: Table S1.** Structure of fused plasmids with different linkers.

**Additional file 5: Table S2.**The primers used for SOE-PCR.

**Additional file 6: Figure S4.** HPLC spectra. (a) Spectra of the (*R*)-3,5-BTPE standard, (b) the 3,5-BTAP substrate, and (c) the sample which the 3,5-BTAP was reduce by bioreaction.

**Additional file 7: Figure S5.** Chiral HPLC spectra. (a) Spectra of the 3,5-BTAP substrate, (b) the (*R*)-3,5-BTPE standard, (c) the (*S*)-3,5-BTPE standard, and (D) the sample which the 3,5-BTAP was reduce by bioreaction.

## Abbreviations

(*R*)-3,5-BTPE: (*R*)-[3,5-bis(trifluoromethyl)phenyl] ethanol
3,5-BTAP: 3,5-bis(trifluoromethyl) acetophenone
NADH: nicotinamide adenine dinucleotide
NADPH: nicotinamide adenine dinucleotide phosphate
*Lk*CR: carbonyl reductase from *Lactobacillus kefir*
*Bs*GDH: glucose dehydrogenase from *Bacillus subtilis*
