# Supplementary figures and images for: Carbonyl reductase identification and development of whole-cell biotransformation for highly efficient synthesis of (R)-[3,5-bis(trifluoromethyl)phenyl] ethanol
Source: Microb Cell Fact. 2016 Nov 11;15:191. doi: 10.1186/s12934-016-0585-5 (PMC5106766; doi:10.1186/s12934-016-0585-5)

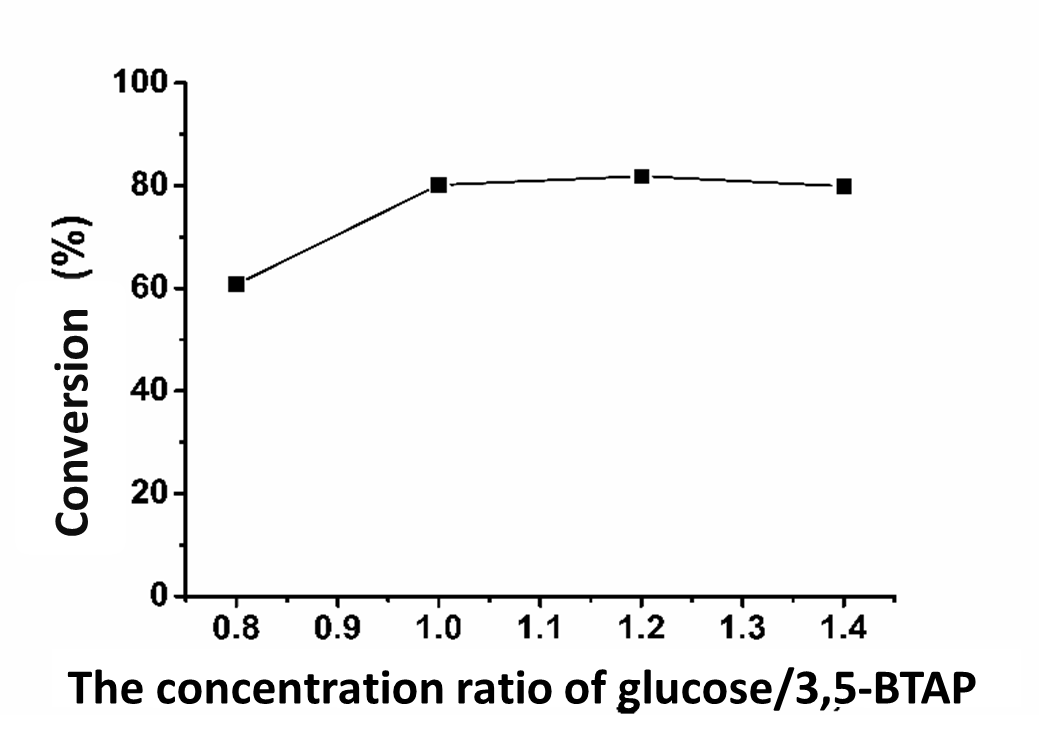

Supplement: Supplementary file 2 — Additional file 2: Figure S2. The effect of glucose concentration on the asymmetric synthesis of (R)-3,5-BTPE using E. coli/pET-BsGDH-LkCR. Reaction conditions: 300 mM substrate, 330-540 mM glucose, 250 g/L wet cells, and incubated at 28°C for 24 h at pH 5.5 with shaking at 220 rpm. [file 12934_2016_585_MOESM2_ESM.tif]

a

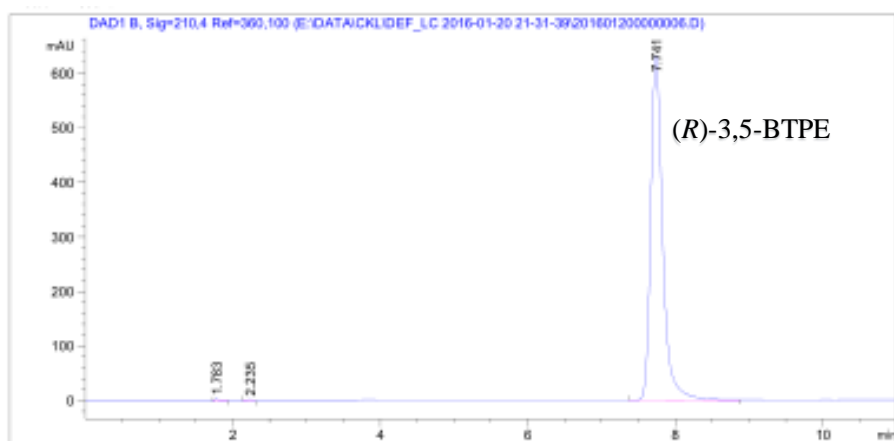

b

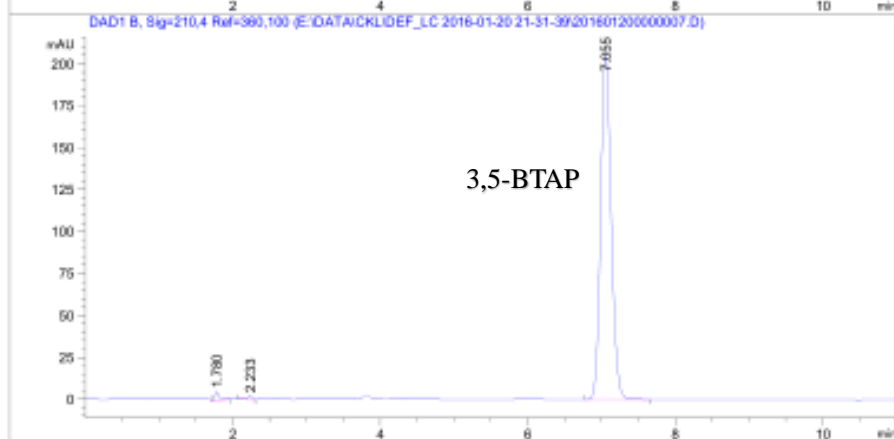

c

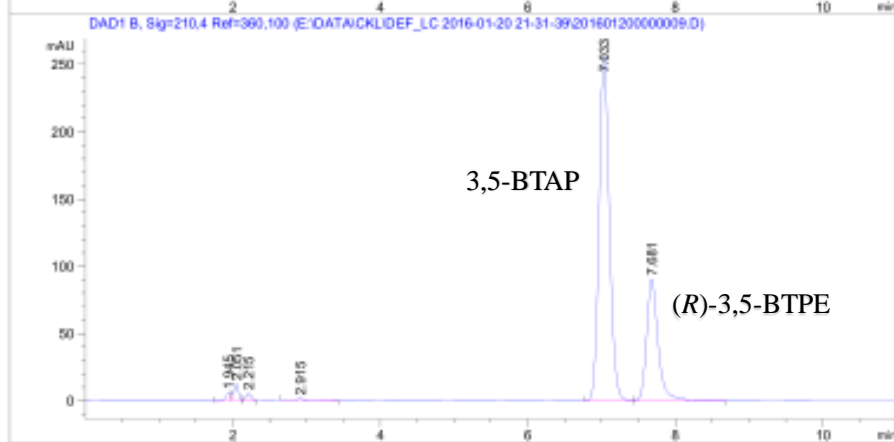

Supplement: Supplementary file 6 — Additional file 6: Figure S4. HPLC spectra. (a) Spectra of the (R)-3,5-BTPE standard, (b) the 3,5-BTAP substrate, and (c) the sample which the 3,5-BTAP was reduce by bioreaction. [file 12934_2016_585_MOESM6_ESM.pdf]

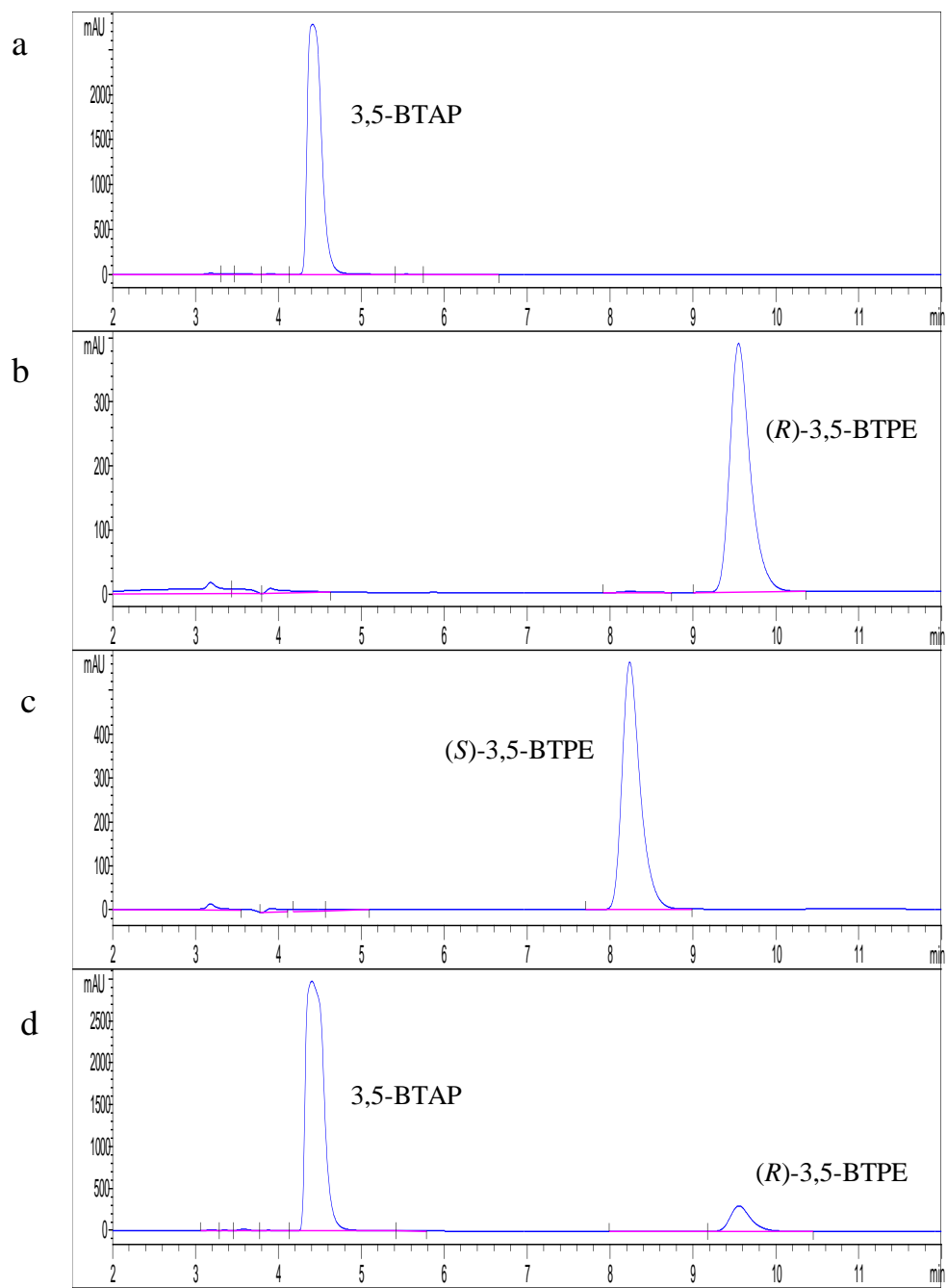

Supplement: Supplementary file 7 — Additional file 7: Figure S5. Chiral HPLC spectra. (a) Spectra of the 3,5-BTAP substrate, (b) the (R)-3,5-BTPE standard, (c) the (S)-3,5-BTPE standard, and (D) the sample which the 3,5-BTAP was reduce by bioreaction. [file 12934_2016_585_MOESM7_ESM.pdf]
