# Supplementary material for: Carbonyl reductase identification and development of whole-cell biotransformation for highly efficient synthesis of (R)-[3,5-bis(trifluoromethyl)phenyl] ethanol
Source: Microb Cell Fact. 2016 Nov 11;15:191. doi: 10.1186/s12934-016-0585-5 (PMC5106766; doi:10.1186/s12934-016-0585-5)
Supplement: Supplementary file 4 — Additional file 4: Table S1. Structure of fused plasmids with different linkers. [file 12934_2016_585_MOESM4_ESM.docx]

**Supplementary Table S1.** **Structure of fused plasmids with different linkers**

| Plasmids | Upstream | linker peptide | Downstream |
| --- | --- | --- | --- |
| pET-*Bs*GDH-GGGGS- *Lk*CR | GDH | GGGGSGGGGSGGGGS | *Lk*CR |
| pET-*Bs*GDH-EAAAK- *Lk*CR | GDH | EAAAKEAAAKEAAAK | *Lk*CR |
| pET-*Bs*GDH-ER/K(5nm) - *Lk*CR | GDH | KAKLKEEEERKQREEEERIKRLEELAKRKEEERK | *Lk*CR |
| pET-*Bs*GDH-ER/K(10nm) - *L*kCR | GDH | EEEEKKKQQEEEAERLRRIQEEMEKERKRREEDEERRRKEEEERRMKLEMEAKRKQEEEERKKREDDEKRKKK | *Lk*CR |
