# Supplementary material for: Carbonyl reductase identification and development of whole-cell biotransformation for highly efficient synthesis of (R)-[3,5-bis(trifluoromethyl)phenyl] ethanol
Source: Microb Cell Fact. 2016 Nov 11;15:191. doi: 10.1186/s12934-016-0585-5 (PMC5106766; doi:10.1186/s12934-016-0585-5)
Supplement: Supplementary file 5 — Additional file 5: Table S2.The primers used for SOE-PCR. [file 12934_2016_585_MOESM5_ESM.docx]

**Supplementary Table S2.** **The primers used for SOE-PCR**

| Plasmid | Forward prime (5’-3’) | Reversed prime (5’-3’) |
| --- | --- | --- |
| pET-GDH-GGGGS- *Lk*CR | CGCGGATCCATGTATCCGGATTTAAAA  GGTGGTGGTGGTAGCGGTGGTGGTGGTAGCGGTGGTGGTGGTAGCATGACTGATCGTTTAAAAGGC | CCGCTCGAGTTATTGAGCAGTGTATCC  CCGCTCGAGTTATTGAGCAGTGTATCC |
| pET-GDH-EAAAK- *Lk*CR | CGCGGATCCATGTATCCGGATTTAAAA  GAAGCAGCAGCAAAAGAAGCAGCAGCAAAAGAAGCAGCAGCAAAAATGACTGATCGTTTAAAAGGC | CCGCTCGAGTTATTGAGCAGTGTATCC  CCGCTCGAGTTATTGAGCAGTGTATCC |
| pET-GDH-ER/K(5nm) - *Lk*CR | CGCGGATCCATGTATCCGGATTTAAAA  TTCCAGGCAGGCCGCGGTAAAGCGAAACTGAAAGAGGAGGAAGAGCGTAA  CGCGGATCCATGTATCCGGATTTAAAA  AAAGAAGAGGAACGCAAAATGACTGATCGTTTAAAAGGCAAAGTAGCAAT  CGCGGATCCATGTATCCGGATTTAAAA | CTCTTTCAGTTTCGCTTTACCGCGGCCTGCCTGGAATGAAGGATATTGTG  TTTGCGTTCCTCTTCTTTACGTTTCGC  TTTTAAACGATCAGTCATTTTGCGTTCCTCTTCTTTACGTTTCGCCAGTT  CCGCTCGAGTTATTGAGCAGTGTATCC  CCGCTCGAGTTATTGAGCAGTGTATCC |
| pET-GDH-ER/K(10nm) - *Lk*CR | CGCGGATCCATGTATCCGGATTTAAAA  TTCCAGGCAGGCCGCGGTGAAGAAGAGGAAAAAAAGAAACAGCAGGAAGA  CGCGGATCCATGTATCCGGATTTAAAA  GAAAAACGTAAGAAAAAAATGACTGATCGTTTAAAAGGCAAAGTAGCAAT  CGCGGATCCATGTATCCGGATTTAAAA | CTTTTTTTCCTCTTCTTCACCGCGGCCTGCCTGGAATGAAGGATATTGTG  TTTTTTCTTACGTTTTTCATCATCCTCG  TTTTAAACGATCAGTCATTTTTTTCTTACGTTTTTCATCATCCTCGCGCT  CCGCTCGAGTTATTGAGCAGTGTATCC  CCGCTCGAGTTATTGAGCAGTGTATCC |
