# Supplementary material for: Carbonyl reductase identification and development of whole-cell biotransformation for highly efficient synthesis of (R)-[3,5-bis(trifluoromethyl)phenyl] ethanol
Source: Microb Cell Fact. 2016 Nov 11;15:191. doi: 10.1186/s12934-016-0585-5 (PMC5106766; doi:10.1186/s12934-016-0585-5)
Supplement: Supplementary file 1 — Additional file 1: Figure S1. Amino acid sequence alignment of CR-1 (GenBank accession number: CP012832.1), CR-2 (AY267012.1), CR-3 (EU877965.1), and CR-4 (CP003119.1). The alignment was performed with the program DNAMAN (http://www.lynnon.com/). Gaps in the aligned sequences are indicated by dots. Highly similar residues are colored in red and framed in blue, while identical residues are in white and on a red background. [file 12934_2016_585_MOESM1_ESM.pdf]

```
                                             1         10        20
CR-1        .................................MASMTGGQQ.MGRGSMLSLGLED....
CR-2        .........................MTDRLKGKVAIVTGGTLGIGLAIADKFVEEG....
CR-3        MKSTIFVKPGKVEIQNIDKPTIQADDDAILHIVRACVCGSDLWAYRDLEDKEPNSENTGH
CR-4        ......................MSSWAELLKGKVAVVTGASRGLGRADALALAEAG....
consensus                                     A   G

                     30        40                60              60
CR-1        ...KVIVVTGGNRGIGAAIVKLLQEMG........AKVAFTDLATDGG.....NTEALGV
CR-2        ...AKVVITGRHADAGEKAAKSIGGTD............................VIRFA
CR-3        EAIAIVDQVGKNITTVKPGDFVIAPFTHGCGHCAACRAGYEGSCQSHSDNFSAGYQAEYV
CR-4        ...ADVVITDILLESDEESKQTAQKYGPLSQVMQSTKVVYAEKTAEDIR..AMGRRALAL
consensus

              70        80        90        100        110       120
CR-1        VANVTDLESMTAAAAEITDKLGPVYGVVANAG.ITKDNFFPKLTPADWDAVLNVNLKGVA
CR-2        QHDASDEAGWTKLFDTTEEAFGPVTTVVNNAG.IGVSKSVEDTTTEEWRKLLSVNLDGVF
CR-3        RYQHAEWSLVKIPGKPEDYSDGMLNSLLTLADVMATGYHAARVANVKPGDTVVVVGDGAV
CR-4        KMDVTDREQVKEVFARVKEEFGSIDILVNNAGTLDHVSQIEKQNDEFWERDLRVNLTGTY
consensus                        G        A                      V   G

              130       140        150       160            170
CR-1        YSIKPFIEGMYERKAGS.IVAISSISGERGNVGQTNYSATKA.....GVIGMMKSLAREG
CR-2        FGTRLGIQRMKNKGLGASIINMSSIEGLVGDPTLGAYNASKG.....AVRIMSKSAALDC
CR-3        GLCGVIASQMRGASRIIAMSRHEDRQKLATEFGATDIVPERGDEAVAKVMALTNGAGADA
CR-4        NCTKAVWPYMKEQGWGR.IINMSSVAGTLGGFGQASYSATKG.....AVLSFTKSMALEG
consensus            M                                      V

          180         190       200          210        220       230
CR-1        AR..YGVRANAVAPGFIDTEMTLAIR...EDIREKITKEIPFRRFGKPEEIAWAVAFLLS
CR-2        ALKDYDVRVNTVHPGYIKTPLVDDLEG..AEEMMSQRTKTPMGHIGEPNDIAWICVYLAS
CR-3        VLECVGSELSTDTAMKVARPGATVGRVGLPHTKKTDLTNSFYSNLAIAGGPASVTTYDKS
CR-4        AR..YGITVNAIVPGIINTEAFRMGN...PKMNERMIQRTAFRRPGEPEDVANAIVFLCS
consensus                                                          A       S

                240       250
CR-1        PVASSYVTGEVLRVNGAHHT...........................
CR-2        DESK.FATGAEFVVDGGYTAQ..........................
CR-3        VLLKAVLDGDIHPGKVFTKRFTLDEIDDAYQAMAKREAIKSLVVAQK
CR-4        DKAK.YITGIGLNVSGGIELFTF........................
consensus           G
```
